# Fabrication and Evaluation of a Micro(Bio)Sensor Array Chip for Multiple Parallel Measurements of Important Cell Biomarkers

**DOI:** 10.3390/s141120519

**Published:** 2014-10-30

**Authors:** Roy M. Pemberton, Timothy Cox, Rachel Tuffin, Guido A. Drago, John Griffiths, Robin Pittson, Graham Johnson, Jinsheng Xu, Ian C. Sage, Rhodri Davies, Simon K. Jackson, Gerry Kenna, Richard Luxton, John P. Hart

**Affiliations:** 1 Centre for Research in Biosciences, Faculty of Health and Life Sciences, University of the West of England, Bristol, BS16 1QY, UK; E-Mails: goomooxu@googlemail.com (J.X.); simon.jackson@plymouth.ac.uk (S.K.J.); 2 QinetiQ Ltd., Malvern Technology Centre, Malvern, WR14 3PS, UK; E-Mails: Timothy.Cox@uwe.ac.uk (T.C.); rachel.tuffin@merckgroup.com (R.T.); ian.sage@ntu.ac.uk (I.C.S.); rrdavies2@gmail.com (R.D.); 3 Applied Enzyme Technology Ltd., Monmouth House, Mamhilad Park, Pontypool NP4 OHZ, UK; E-Mail: Guido@gwent.org; 4 Uniscan Instruments Ltd., Sigma House, Burlow Rd., Buxton, Derbyshire SK17 9JB, UK; E-Mails: John.Griffiths@uniscan.co.uk (J.G.); Graham.Johnson@uniscan.co.uk (G.J.); 5 Gwent Electronic Materials Ltd., Monmouth House, Mamhilad Park, Pontypool NP4 OHZ, UK; E-Mail: Robin@gwent.org; 6 AstraZeneca R&D, Alderley Park, Macclesfield, SK10 4TF, UK; E-Mail: jgerrykenna@gmail.com; 7 Institute of Biosensing Technology, University of the West of England, Bristol, BS16 1QY, UK; E-Mail: Richard.Luxton@uwe.ac.uk

**Keywords:** microbiosensor, screen-printing, MEMS, 96-well plate, metabolism, amperometry, cell monitoring, toxicity testing

## Abstract

This report describes the design and development of an integrated electrochemical cell culture monitoring system, based on enzyme-biosensors and chemical sensors, for monitoring indicators of mammalian cell metabolic status. MEMS technology was used to fabricate a microwell-format silicon platform including a thermometer, onto which chemical sensors (pH, O_2_) and screen-printed biosensors (glucose, lactate), were grafted/deposited. Microwells were formed over the fabricated sensors to give 5-well sensor strips which were interfaced with a multipotentiostat via a bespoke connector box interface. The operation of each sensor/biosensor type was examined individually, and examples of operating devices in five microwells in parallel, in either potentiometric (pH sensing) or amperometric (glucose biosensing) mode are shown. The performance characteristics of the sensors/biosensors indicate that the system could readily be applied to cell culture/toxicity studies.

## 1. Introduction

The miniaturisation of sensing/biosensing devices is highly desirable in order to facilitate their integration into monitoring and detection systems for cell culture applications where high-throughput multiple analyses are desirable. There is great demand in the pharmaceutical industry for improved methods for toxicity testing of new compounds using approaches that can provide real-time information as to the status of target mammalian cells in culture. Biosensors for glucose and lactate can be used to provide information on the metabolic uptake or output of target cells in response to toxic insult, whilst so-called “house-keeping” sensors for temperature, pH, and dissolved oxygen can be used to monitor the prevailing conditions in the culture medium.

Various approaches have been used to develop chemical sensors to measure changes in pH and O_2_ level, and to develop electrochemical biosensors for glucose and lactate. These have included devices incorporating macroelectrodes operating within flowing bioreactor streams, and those of somewhat smaller dimensions compatible with microfluidic systems and suitable for low-cost point-of-care diagnostic deployment. Li *et al.* [1] have described a lab-on-a-tube approach based on small electrochemical biosensors for lactate, glucose and oxygen which operate in chronoamperometric mode for serum samples. Two publications from groups working on paper-based microfluidic electrochemical systems have described screen-printable biosensor devices capable of quantifying glucose, lactate and uric acid in serum [2], and glucose and heavy metals in aqueous solutions [3] for batch-mode analysis at low cost. A multiwell system for monitoring changes in open-circuit potential by human cells has also been reported, based on miniaturised screen-printed gold electrodes [4]. A metabolite-monitoring system based on microfluidic sampling of culture medium from a flask into an electrochemical biosensor platform for glucose and lactate measurement has been described by Boero *et al.* [5]. Development of an integrated microfluidic microchip system has recently been described which includes a cell cultivation chamber with integrated pH and oxygen measurement capabilities, and downstream glucose and lactate biosensors, for monitoring human cancer cell metabolism [6].

The aim of the work described in the present paper was to develop an electrochemical detection system capable of monitoring house-keeping parameters in real time in mammalian cell culture. This approach differs from those described above in aiming for continuous operation of the sensors/biosensors within quiescent culture medium. In order to address this problem, the challenges lay in fabricating sensors/biosensors of sufficiently small dimensions that they could operate as microelectrodes under diffusion-control without the requirement for stirring. Our previous work [7] on developing screen-printable water-based carbon inks containing both a redox mediator (cobalt phthalocyanine; CoPC) and an enzyme (glucose oxidase; GOx, or lactate oxidase; LOx) showed that glucose [8] and lactate [9] biosensors can be screen-printed and used as microband electrodes of micron size in at least one dimension and thus exhibit steady-state current behaviour. These microband biosensors have been used to make continuous amperometric measurements in tissue culture medium [10,11] when placed in 3 mL volume culture wells using a laboratory apparatus. Although this approach was not suited to a high throughput scenario, the potential to use these screen-printed microbiosensors in real-time toxicity testing was demonstrated by their ability to monitor changes in the uptake of glucose [12] or the production of lactate [11] by mammalian cells in the presence of selected toxins.

The goal of obtaining a sensor/biosensor system for high-throughput screening of cell culture housekeeping parameters led us to design a microstructured sensor/biosensor array located at the base of a microwell. The design and fabrication of the required microsensors involved the use of a micro-electro-mechanical systems (MEMS) fabricated silicon platform onto which chemically specific electrode functionalities could be added; these included screen-printed miniaturised biosensor microelectrodes for glucose and lactate, and chemical microelectrode sensors for pH and O_2_; a temperature sensor was also incorporated. The fabrication of these devices onto a silicon substrate base in a 96-well format and the evaluation of their performance characteristics are described in this paper.

## 2. Experimental Section

### 2.1. Silicon Wafer Microstructure Design

**Figure 1.**
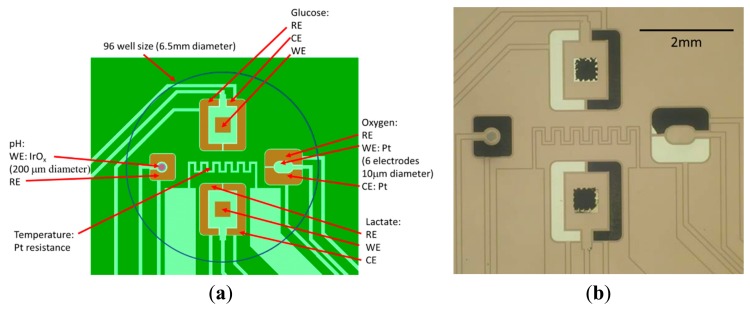
(**a**) Scale diagram of integrated well design for microstructured sensor/biosensor chip base showing locations of four sensors and Pt resistance thermometer in a single 96-well format; (**b**) Photograph of part of the completed 5-well strip showing the sensors printed within the region of a single 96-well.

Microstructures were fabricated on 100 mm diameter, 0.5 mm-thick Si wafers in the form of platinum tracks and exposed pads using MEMS based optical lithography and dry etching processes. The design was such that Pt pads were left exposed for the subsequent fabrication of sensors/biosensors and their respective counter/reference electrodes. Figure 1a shows the microstructure design for a single microwell: insulated Pt tracks are shown (light green), including the insulated Pt temperature sensor, together with schematic representations (brown) of the fabricated active sensing areas for pH, O_2_, glucose and lactate sensors/biosensors, plus their respective reference and counter electrodes. The completed electrode structure within a 6.5 mm diameter circle, *i.e.*, a 96 well format, is shown in Figure 1b.

**Figure 2.**
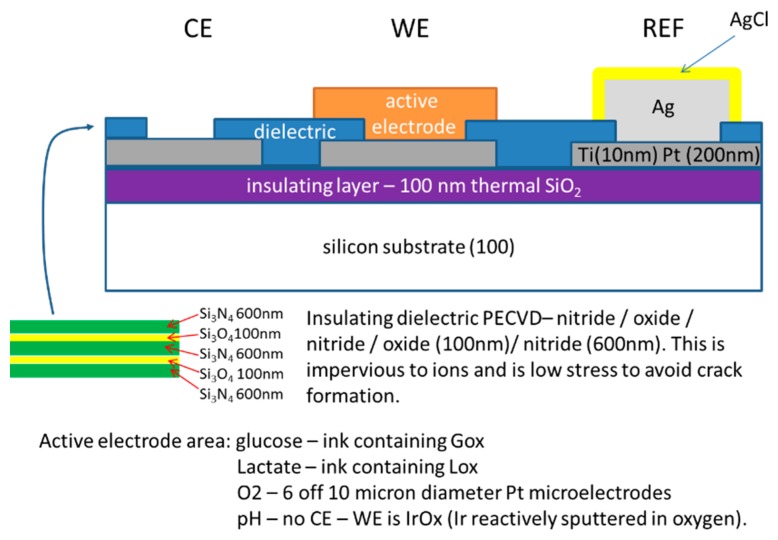
Diagram illustrating cross-section (not to scale) through the MEMS-microfabricated sensor device platform.

The basis for the fabrication of all of the sensors is illustrated in Figure 2, which shows a generalized diagrammatic representation of a single sensor within the microfabricated platform in cross-section. The underlying SiO_2_-coated silicon substrate is shown, upon which a titanium (10 nm)/platinum (100 nm) was deposited by sputtering. This layer is then patterned into tracks using optical lithography and reactive ion etching using fluorine based chemistry (CF_4_) [13,14]. Alignment marks were etched into the platinum for the alignment of future lithography steps, and also of the screen-printed layers.

The key feature in the fabrication of the generic platform for the different sensors was the insulating silicon nitride/silicon dioxide multilayered dielectric structure (obtained by plasma-enhanced chemical vapor deposition; PECVD), which coated the Pt layer; this insulating layer was impervious to ions and had good adhesion to the underlying metallization. The insulating layer was then patterned using lithography and reactive ion etching in a CHF_3_/O_2_ mixture to leave exposed Pt pads, onto which the active layer for each sensor/biosensor was then grafted (screen-printed/or deposited) to produce the response specific to each of the target analytes. The Pt resistor for temperature measurement remained buried beneath the silicon dioxide/silicon nitride sandwich.

The microstructure footprint for a single microwell was a 6.5 mm-diameter circle which would form the base of a 96-well microplate-sized well. Five replicate well designs were fabricated onto a single Si wafer strip measuring 71 mm × 16 mm (Supplementary Material, Figure S1). Insulated Pt tracks ran from each electrode to one long edge of the 5-well strip to be used for interfacing with the potentiostat via a bespoke edge connector.

### 2.2. Ag/AgCl Reference Electrodes

A dedicated Ag/AgCl reference electrode was prepared in turn for each sensor/biosensor (O_2_, pH, glucose, lactate). These were prepared by electroplating silver from a solution containing 0.2 M silver nitrate, 2 M potassium iodide, 0.5 mN sodium thiosulfate on the appropriate Pt pad areas at a current density of 20 mA/cm^2^ for 5 min. The silver was then chloridised in 0.1 M HCl for 30 s to produce a silver chloride overlayer.

### 2.3. Temperature Sensor

This was a single microfabricated Pt track of square waveform design (see Supplementary Material, Figure S2a). The track was of width 75 μm and length total 700 μm.

### 2.4. pH Sensor

**Figure 3.**
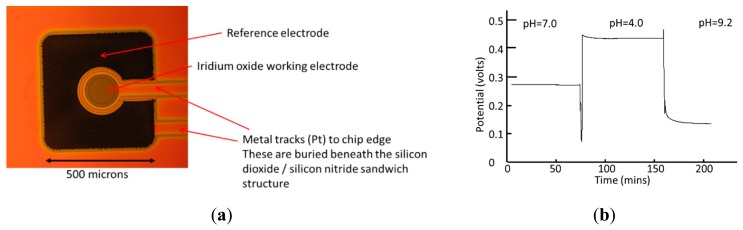
pH sensor: (**a**) Photograph of microfabricated iridium oxide (IrOx) sensor (**b**) Potential-time response following substitution of different value pH buffer solutions in microwell.

The pH sensor was fabricated as a 2-electrode potentiometric device (Figure 3a). Initially, electrodeposited iridium oxide (IrO_x_) layers were investigated as the pH sensitive surface. These gave a good pH response but had poor adhesion to the underlying platinum pad and consequently had a short lifetime in culture media. The final pH sensing surface was fabricated from iridium oxide which was deposited by reactive sputtering of iridium in an oxygen containing plasma (Teer Coatings, Droitwich, UK). The iridium oxide layer was patterned by a lift off technique. The potential of this surface was measured against an electroplated Ag/AgCl reference electrode to form the complete pH sensor.

### 2.5. O_2_ Sensor

**Figure 4.**
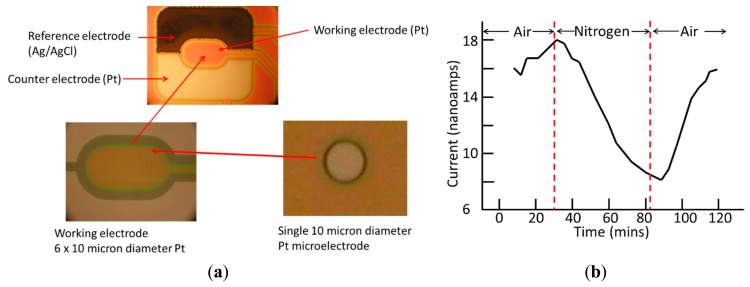
O_2_ sensor: (**a**) Photograph of microfabricated oxygen sensor showing bare platinum working and counter electrodes; working electrodes = 6 off 10 micron diameter. (**b**) Amperometric response of (collagen-coated) oxygen sensor to nitrogen purging; and recovery.

O_2_ sensors comprised 3-electrodes: a working Pt microelectrode array (six 10 μm diameter discs), an electroplated Ag/AgCl reference electrode (1500 × 400 μm), and a Pt counter electrode (1500 × 400 μm) (see Figure 4a).

The six Pt microelectrodes were exposed by lithography followed by reactive ion etching through the silicon nitride/silicon dioxide sandwich structure. The optimum diameter for the Pt microelectrodes was determined from preliminary experiments using test chips fabricated with eight individually addressable Pt microelectrodes, with diameters of 5, 10, 20, 50, 75, 100, 200 and 300 μm. For the fabrication of these test structures, the overlayer dielectric used was the photodefinable epoxy SU8 (MicroChem Corp., Westborough, MA, USA). The eight microelectrodes of different diameters were tested in a 3-electrode system in potassium ferrocyanide solution using both cyclic voltammetric and amperometric measurement methods. Cyclic voltammetry (the shape of the voltammograms) was used to determine that behaviour was steady-state and indicative of microelectrode behaviour, rather than a planar-diffusion-controlled response. Amperometry was then used to determine whether a steady-state current response could be sustained. The final choice of a 10 μm diameter electrode was arrived at since this was the minimum diameter electrode satisfying both of these criteria, whereas the smaller 5 μm diameter electrodes did not give reproducible current responses. The ability to produce a steady-state amperometric response was then confirmed using an oxygenated phosphate buffer solution. The resulting O_2_ sensors were tested using linear sweep voltammetry to determine the optimum applied voltage for the response to O_2_ in an oxygenated phosphate buffer between 0 V and −1.5 V.

### 2.6. Biosensors (Glucose and Lactate)

**Figure 5.**
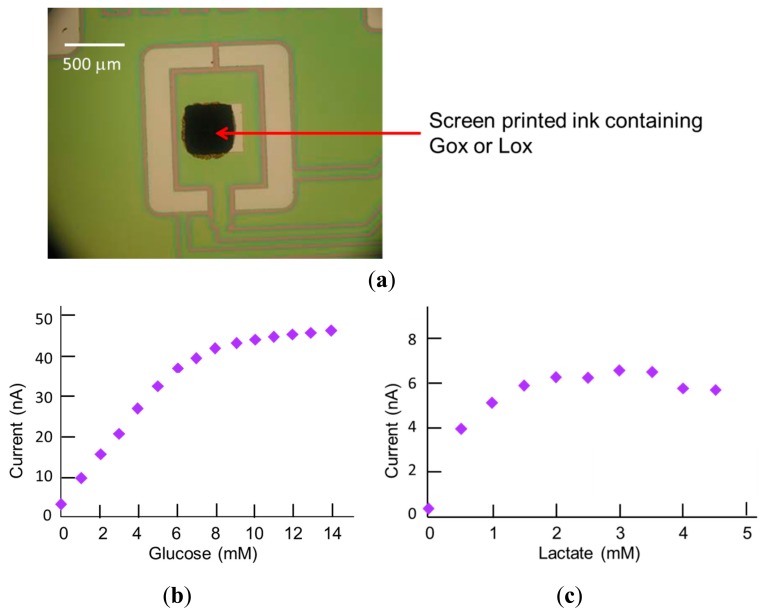
Enzyme biosensors: (**a**) Photograph of microfabricated screen-printed microbiosensor showing screen-printed working electrode ink deposit (**b**) Calibration plot for glucose biosensor in phosphate buffer (**c**) Calibration plot for lactate biosensor in phosphate buffer.

The glucose and lactate microbiosensor working electrodes were screen-printed using water-based carbon ink formulations containing CoPC and either GOx (product code C2041124D3, GEM, Pontypool, UK) [15] or LOx (C2080519D3) [9]. The ink formulations for these working electrodes were screen-printed directly onto Pt electrodes on the MEMS chip in a 500 × 500 μm square pattern, situated in the centre of a rectangular area bordered by the Pt counter and previously electroplated Ag/AgCl reference electrodes (Figures 1 and 5a). The screen for printing was aligned to marks present within the platinum layer on the MEMS chip.

### 2.7. Culture Well Construction

Once the sensors/biosensors had been fabricated on the Si MEMS chip, wells were formed by attaching strips of five bottomless 96-well format strips (Greiner Bio-one Ltd., Gloucestershire, UK) to the Si bases using 0.5 mm-thick press-to-seal 6.5-mm diameter hole silicone adhesive strips (Grace Bio-Labs Inc., Bend, OR, USA). Completed 5-well sensor strips are shown in the Supplementary Material, Figure S3. Prior to experimentation with mammalian cells, the bases of the microwells were coated with collagen, as described elsewhere [16], in order to facilitate cell adhesion. Antibiotics (penicillin/streptomycin) were included in the medium to prevent bacterial growth.

### 2.8. Instrumentation

**Figure 6.**
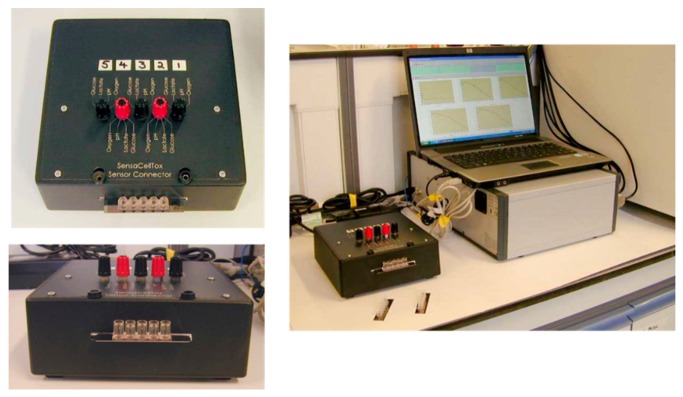
Photographs of (left hand images)—5-well sensor strip interfaced with connector box; and (right hand image)—connection to multi-potentiostat workstation. The response of the five sensors can be seen on the display screen.

The 5-well sensor well strips were connected, via a custom-made interface box, to a PG580RM 5-channel potentiostat (Uniscan Instruments Ltd, Buxton, UK) (Figure 6) which was controlled using UiEChem software (Version 2.02, Uniscan Instruments). The pitch of the platinum tracks on the edge of the MEMS chip was matched to that of an edge connector in the interface box—the chip was sandwiched against the edge connector via a gold Zebra^®^ strip. The potentiostat enabled the simultaneous application of five sensors using either potentiometric or amperometric techniques. The interface box incorporates switches that allow the independent selection among the five sensors for use within each well.

In order to maintain the well at a temperature suitable for cell culture, the sensor well strips and connector box were placed in a non-humidified 37 °C incubator, and were connected to the potentiostat which remained outside the incubator. The mammalian cells used in the study were human choriocarcinoma (BeWo) cells; these are adherent cells, known for their high glycolytic activity. Cells were placed into microwells in 300 microlitres of culture medium containing 2 mM glutamine and 10% (v/v) foetal calf serum. The microwells were capped with loose-fitting polyester caps to minimise evaporation whilst allowing gas exchange.

## 3. Results and Discussion

### 3.1. Performance Characteristics of Sensors/Biosensors

#### 3.1.1. Ag/AgCl Reference Electrodes

The potential of the plated Ag/AgCl reference electrodes was stable within ±2 mV with respect to a commercial reference electrode over a 24 h period. The cyclic voltammograms of 10^−4^ M [Fe(CN)_6_]^3−/4−^ at a screen-printed glucose biosensor working electrode in a mixture of 0.05 M sodium phosphate buffer (pH 7.2) and 50 mM NaCl as supporting electrolyte, showed redox peaks at +356 mV and +350 mV *vs.* the plated Ag/AgCl reference electrode. These results showed an offset of ≤ 10 mV compared to those obtained using a commercial Ag/AgCl reference electrode.

#### 3.1.2. Temperature Sensor

The platinum resistance sensor had a typical resistance of 152 Ω at 20 °C. The variation of resistance was linear with temperature over the range 20–60 °C (Figure S2b). The slope was +0.2 Ω/°C, corresponding to a temperature coefficient for resistivity of 1.4 × 10^−3^·°C^−1^ (value for bulk Pt is 3.9 × 10^−3^·°C^−1^) [17]. This value is similar to (1440 ± 100) × 10^−6^·°C^−1^, previously reported for a sputtered thin film platinum resistance thermometer [18].

#### 3.1.3. pH Sensor

The pH sensors were evaluated using phosphate buffer at pHs 4.0, 7.0 and 9.2. The five pH sensors in a 5-well chip were connected to separate channels of the multipotentiostat, then the wells were filled with 300 μL of the appropriate buffer. Rapid responses (<1 min) to changing pH were observed, which reached true steady-state potential values within 10 min (Figure 3b) and showed a drift of less than 0.01 pH units per hour. The pH changes per well for a given change in pH were constant. For five replicate sensors, a mean slope of −58.5 mV/pH unit (CV of 0.97%) was obtained. The performance and stability achieved was superior for the reactively sputtering iridium oxide layer as compared with the electroplated layers. The latter resulted in a low stability surface with poor adhesion, resulting in high rates of drift.

#### 3.1.4. O_2_ Sensor

The performance of the O_2_ sensor was assessed using a pH 7.3 phosphate buffer. The choice of applied potential was based on the results of linear sweep voltammetric scans in an oxygenated buffer solution. The resulting voltammograms showed a cathodic current response to oxygen that began at around −0.2 V and became increasingly negative, rising to a plateau between −0.5 V and −0.9 V. A value of −0.6 V, being a value close to the beginning of this plateau, and where the current is under diffusion control, was therefore selected as the optimum applied potential for making oxygen measurements. O_2_ concentration in the solution was varied by purging the solution with N_2_ using a fine needle attached to a gas line. Sensitivity and speed of response were then assessed after the solution was equilibrated with air. Initial experiments in a bulk solution on large (200 μm diameter) sensors were used to test the procedure. This large diameter sensor responded well to the removal of oxygen by nitrogen purging, and showed good reversibility (Supplementary Material, Figure S4). When experiments were performed on the 10 μm diameter sensors in a 300 μL buffer volume inside the microwells (purged using a small needle on a nitrogen line), responses to removal of oxygen were observed and were reversible over one cycle, but a baseline drift was observed. The drifting baseline indicated a degradation of the platinum surface which was more significant for the smaller 10 micron diameter sensor(s). Interestingly, when microwell surfaces were coated with a collagen layer as an optional pre-treatment conditioning for experiments with live mammalian cells, the response of the O_2_ sensor was gradually restored (Figure 4b).

#### 3.1.5. Glucose and Lactate Biosensors

An example of a screen-printed working enzyme-based electrode ink is shown in Figure 5a. In some instances there was some misalignment of the deposited ink on the Pt pad; however, as explained in a previous publication [16], this did not degrade the biosensor performance provided that a good electrical contact is made between the ink and the Pt pad. Calibration plots for glucose and for lactate were obtained from experiments conducted in phosphate buffer, pH 7.3, with incremental additions of their respective analyte. The electrodes were operated in amperometric mode at an applied potential, based on previous studies [16], of +0.4 V *vs.* Ag/AgCl. The sensitivity of the glucose microbiosensor (Figure 5b) was about 6 nA/mM and linear up to 5 mM, with a working range of around 10 mM. The response of the lactate microbiosensor (Figure 5c) was of similar sensitivity (5 nA/mM), but the working range was only up to 1 mM. Under cell culture conditions, initial glucose concentrations are usually 10 or 25 mM, depending on whether medium is high- or low-glucose-containing. In tissue culture studies, glucose uptake by BeWo cells has been shown to exhibit Michaelis-Menten kinetics and occurs most rapidly for glucose concentrations of up to 15 mM, consistent with low glucose medium, while saturation occurs at around 20 mM [19]. The working range observed for glucose microbiosensors in uncoated wells would therefore be too short to measure initial glucose uptake by cells, even in low glucose medium. However, as reported previously [16], and described below, the effect of coating wells (and therefore biosensors) with collagen is similar to the effect of adding a membrane layer, and this results in a lengthening of the working range of the glucose biosensors to around 10 mM, which renders the biosensors suitable for measuring glucose uptake from low glucose-containing medium. The same is true of the lactate biosensors and would predict an extension of the working range up to around 2 mM after collagen coating; this level would be sufficient in the case of lactate. Studies by other workers [20] using trophoblast cells have shown that during the first 6 h of incubation, lactate concentrations in control cultures rise to around 1.5 mM, whereas under hypoxic conditions, levels can reach 4 mM. This type of example suggests that the lactate biosensors would be suitable for giving an early warning of cell hypoxia or other alteration in metabolism affecting lactate output.

### 3.2. Operation of Multiple Sensors/Biosensors and Mammalian Cells

As a demonstration of the complete integrated system, the following results represent one example of five sensors run in potentiometric mode (pH sensors) and one example of five sensors run in amperometric mode (glucose biosensors).

#### 3.2.1. Potentiometric Sensors: pH Sensing

**Figure 7.**
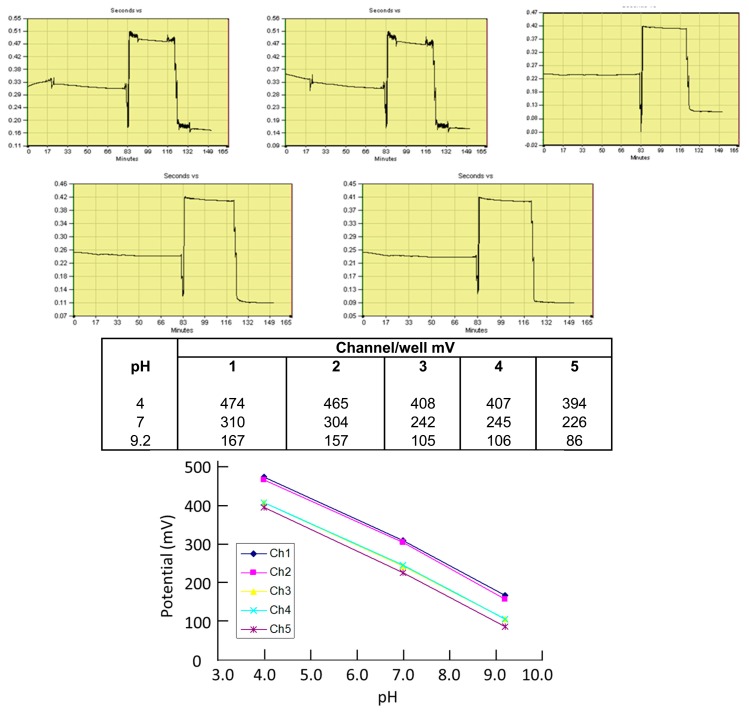
Screen-capture showing response of five pH sensors run in parallel in collagen-coated wells containing standard pH buffer solutions at pHs 4.0, 7.0 and 9.2. Table shows steady-state potential at each pH; graph shows slopes of potential *vs.* pH for each sensor.

Figure 7 shows the results obtained for five pH sensors (one in each well) run in parallel in which pH 4.0, 7.0 and 9.2 buffer was introduced in turn. These runs were conducted in wells which had been coated with collagen to provide a surface that could encourage cell adhesion if required. There was an offset in voltage between wells, however the pH dependence of the potential was the same in each well, giving a slope of −54 mV/pH unit (CV = 1%). Drift on collagen-coated wells was 0.01 pH unit/hour. In operation, any differences in starting voltage between pH sensors would be normalized to allow direct comparison between different wells in real time.

Individual pH sensors in collagen-coated wells were also tested over 24 h in the presence of living mammalian cells. Potentiometric responses showed increasing readings with time which correlated with the drop in pH which accompanies cell growth and the production of lactate into the cell culture medium. The starting pH was 7.2 (170 mV) and remained the same up to 3000 s; at 5000 s the pH had decreased to 7.1 (190 mV). Further decreases in pH were as follows: 7000 s (pH 7.0, 210 mV), 8000 s (pH 6.9, 220 mV) and 9000 s (pH 5.3, 320 mV).

#### 3.2.2. Glucose Sensing

**Figure 8.**
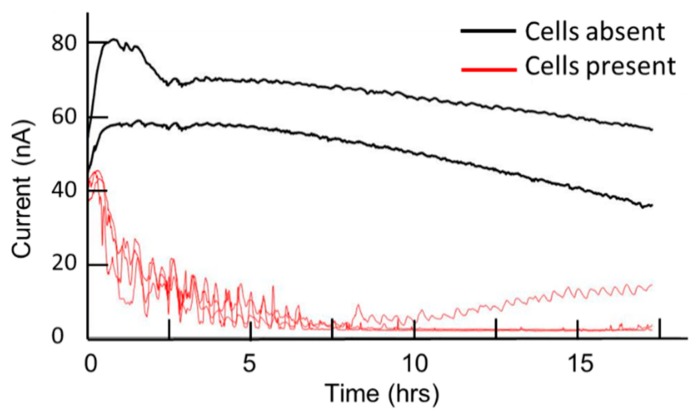
Amperometric responses obtained for five collagen-coated glucose microbiosensors run over 24 h. Wells contained 340 μL of culture medium in the absence or the presence of 2 × 10^5^ BeWo cells. E_app_ = +0.4 V; Temp = 37 °C; 5% CO_2_ dry.

Five microwells containing glucose microbiosensors were run in parallel in amperometric mode (E_app_ = +0.4 V). The wells were collagen-coated and contained culture medium with added glucose. Three of the wells contained 2 × 10^5^ mammalian cells, while the remaining two control wells contained only culture medium. Figure 8 shows the responses obtained for the five microbiosensors. From a starting current response of around 70 nA for all five biosensors, those in the wells containing cells showed a rapid decrease in response over the first 2 h, which continued decreasing to baseline level by 8 h. In contrast, the two wells where cells were absent retained current responses of 50–70 nA at 15 h. These results are consistent with the known high glycolytic activity of the cells, which results in a rapid uptake of glucose from the culture medium, and its complete removal after 8 h in culture. This speed of glucose uptake compares with 9 h reported previously [12] for hepatocytes seeded at higher density (10^6^/mL) and may reflect the known high metabolic rate of the BeWo cells used in the present study.

## 4. Conclusions/Outlook

As the basis for micro-sensor/biosensor fabrication, a silicon substrate was chosen as it permitted the use of MEMS microfabrication techniques to deposit robust layers such as platinum metallisation and inorganic dielectrics such as silicon nitride and silicon dioxide. These proved to be impervious to electrolytes used in the cell culture environment. Five sensor types were fabricated onto this base: the temperature, pH, glucose and lactate devices all showed performance characteristics consistent with proof-of-principle for eventual use in cell culture monitoring. The use of microelectrodes means that the electrochemically-based measurement process, particularly in the case of glucose and lactate measurements, does not perturb the chemical composition of the culture medium. The O_2_ sensor in its present format displayed a drifting baseline which may be due to surface oxidation or fouling. Further development of this sensor is therefore required to build in reversibility and the possibility for continuous measurement.

The testing of five sensors in parallel, either in potentiometric mode (pH) or amperometric mode (glucose, lactate) shows promise; in terms of outlook, the number of replicate wells and measurements could be scaled up for higher throughput analysis. Underlying this assumption would be the aim of further integrating the electronics with the cell culture plate(s) to enhance portability and robust operation. The MEMS sensor platform described herein could be interfaced directly with silicon-based signal conditioning electronics and components, such as miniaturised potentiostats, resulting in a truly integrated smart substrate. An integrated microwell platform comprising an array of electrochemical sensors and biosensors, based around additional enzymes for other analytes, would be valuable in monitoring cell metabolic activity for a variety of application areas, including toxicity testing of candidate drugs in the pharmaceutical industry.

## Supplementary Material
