# Supplementary material for: Fabrication and Evaluation of a Micro(Bio)Sensor Array Chip for Multiple Parallel Measurements of Important Cell Biomarkers
Source: Sensors (Basel). 2014 Oct 30;14(11):20519–32. doi: 10.3390/s141120519 (PMC4279497; doi:10.3390/s141120519)
Supplement: Supplementary file 1 [file sensors-14-20519-s001.pdf]

OPEN ACCESS

sensors

ISSN 1424-8220
www.mdpi.com/journal/sensors

Supplementary Information

Fabrication and Evaluation of a Micro(Bio)Sensor Array Chip for Multiple Parallel Measurements of Important Cell Biomarkers. *Sensors* 2014, *14*, 20519-20532

Roy M. Pemberton [1,*], Timothy Cox [2,†], Rachel Tuffin [2], Guido A. Drago [3], John Griffiths [4], Robin Pittson [5], Graham Johnson [4], Jinsheng Xu [1], Ian C. Sage [2], Rhodri Davies [2], Simon K. Jackson [1,‡], Gerry Kenna [6], Richard Luxton [7] and John P. Hart [1,*]

[1] Centre for Research in Biosciences, Faculty of Health and Life Sciences, University of the West of England, Bristol, BS16 1QY, UK; E-Mails: goomooxu@googlemail.com (J.X.); simon.jackson@plymouth.ac.uk (S.K.J.)

[2] QinetiQ Ltd., Malvern Technology Centre, Malvern, WR14 3PS, UK; E-Mails: Timothy.Cox@uwe.ac.uk (T.C.); rachel.tuffin@merckgroup.com (R.T.); ian.sage@ntu.ac.uk (I.C.S.); rrdavies2@gmail.com (R.D.)

[3] Applied Enzyme Technology Ltd., Monmouth House, Mamhilad Park, Pontypool NP4 OHZ, UK; E-Mail: Guido@gwent.org

[4] Uniscan Instruments Ltd., Sigma House, Burlow Rd., Buxton, Derbyshire SK17 9JB, UK; E-Mails: John.Griffiths@uniscan.co.uk (J.G.); Graham.Johnson@uniscan.co.uk (G.J.)

[5] Gwent Electronic Materials Ltd., Monmouth House, Mamhilad Park, Pontypool NP4 OHZ, UK; E-Mail: Robin@gwent.org

[6] AstraZeneca R&D, Alderley Park, Macclesfield, SK10 4TF, UK; E-Mail: jgerrykenna@gmail.com

[7] Institute of Biosensing Technology, University of the West of England, Bristol, BS16 1QY, UK; E-Mail: Richard.Luxton@uwe.ac.uk

† Current address: Institute of Biosensing Technology, University of the West of England, Bristol, BS16 1QY, UK

‡ Current address: Centre for Research in Translational Biomedicine, School of Biomedical and Biological Sciences, University of Plymouth, Plymouth PL4 8AA, UK

* Authors to whom correspondence should be addressed; E-Mails: Roy.Pemberton@uwe.ac.uk (R.M.P.); John.Hart@uwe.ac.uk (J.P.H.); Tel.: +44-117-328-2469 (J.P.H.); Fax: +44-117-328-2904 (J.P.H.).

Figure S1. Overall view of the design for the 5-well MEMS sensor chip with five sensors per well base (25 sensors in total).

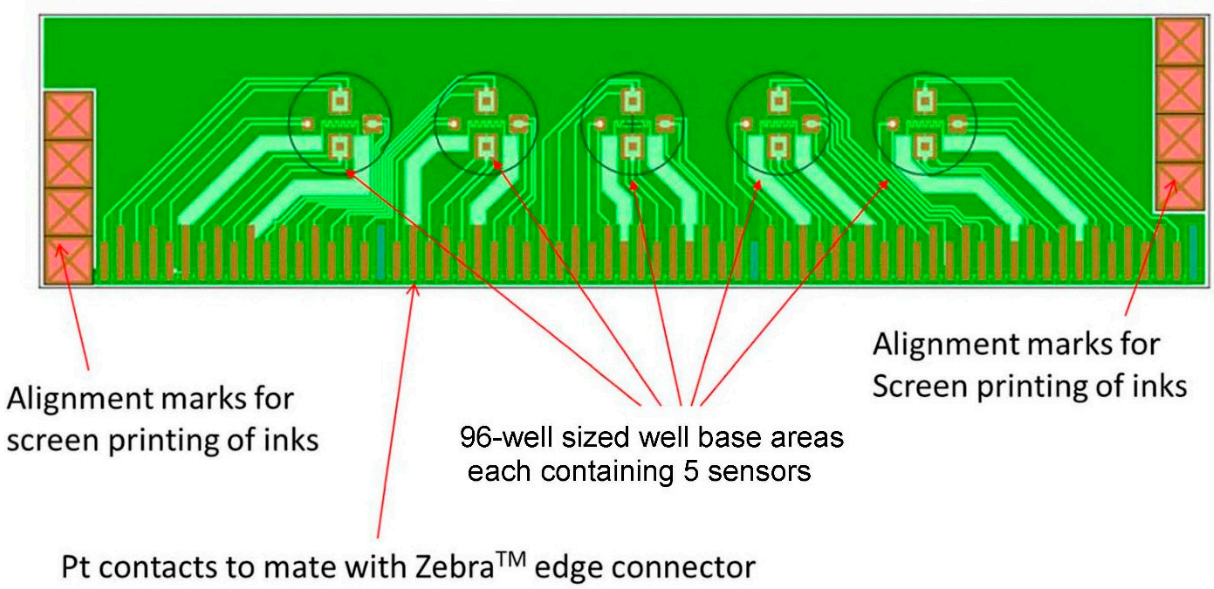

Figure S2. Temperature sensor: (**a**) Photograph of microfabricated Pt resistance track (**b**) Graph of resistance *vs.* temperature.

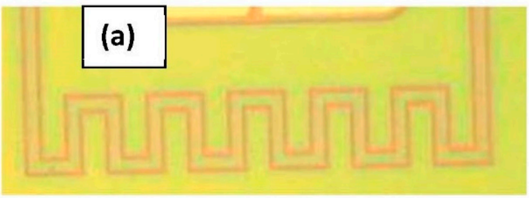

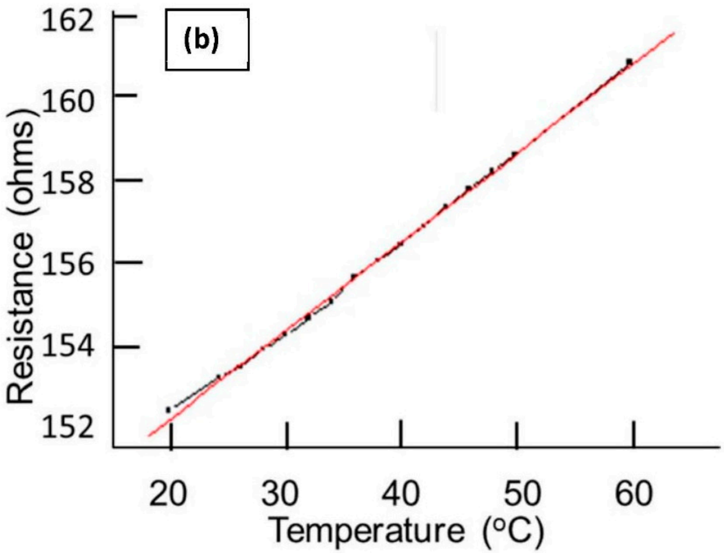

Figure S3. 5-well sensor strips. Bottomless wells have been added to the surface of the MEMS chips. These are now ready for insertion into the connector box, followed by addition of culture medium and cells.

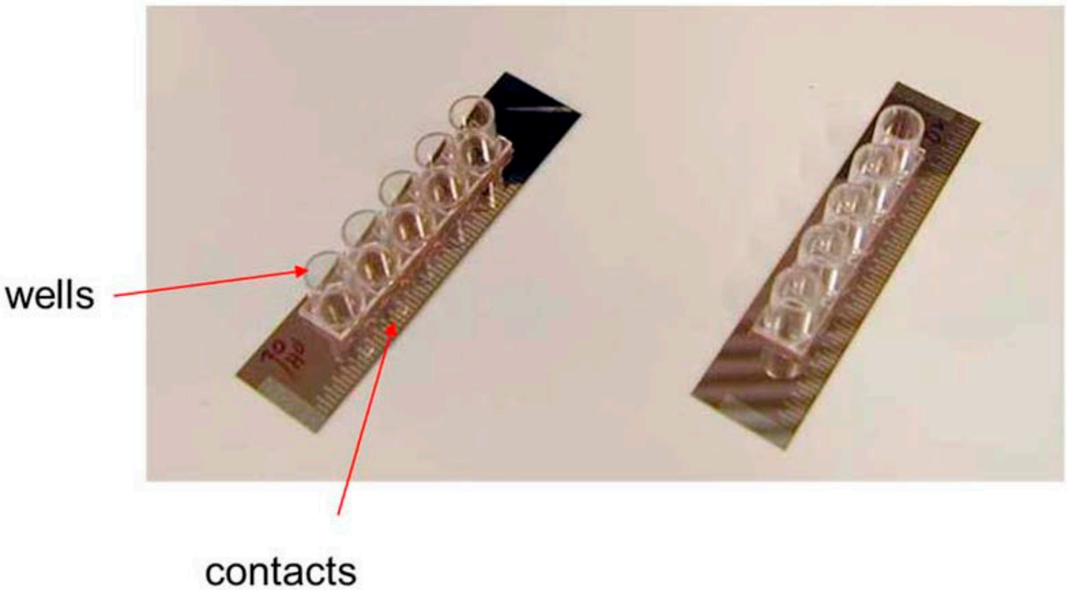

Figure S4. Current responses obtained for a 200 micron diameter oxygen sensor tested in 10 mL bulk solution of phosphate buffer, pH 7.3, versus a commercial Ag/AgCl reference electrode; E_{app} = −0.6 V. Graph shows response upon alternate purging with Nitrogen gas and equilibration with air.

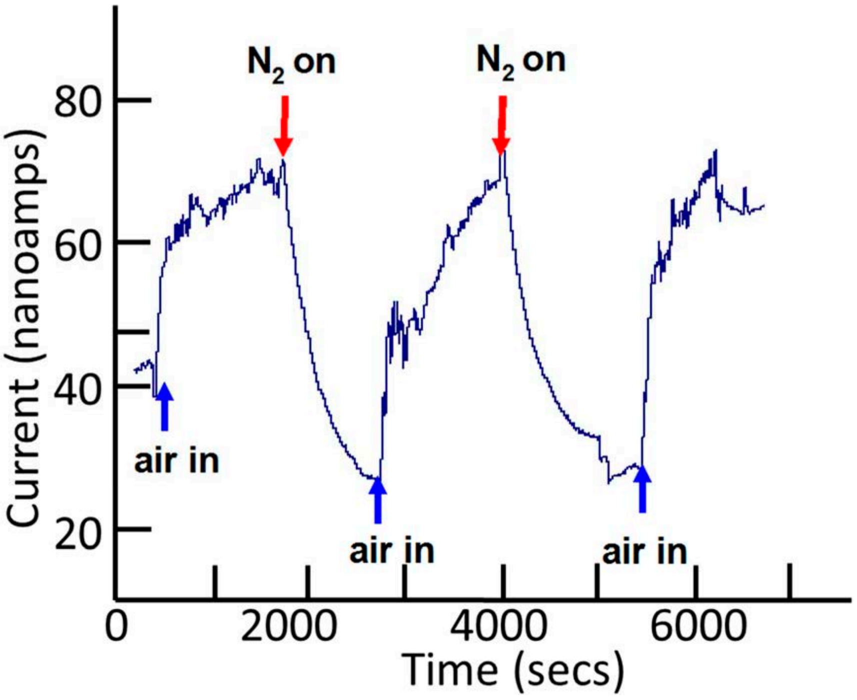

Figure S5. Current responses obtained for a 6 × 10 m diameter oxygen sensor tested in a 96-well sensor strip solution of phosphate buffer, pH 7.3. 3-electrode system; $E_{app} = -0.6$ V. Graph shows response upon alternate purging with Nitrogen gas and equilibration with air. Illustrates drifting baseline.

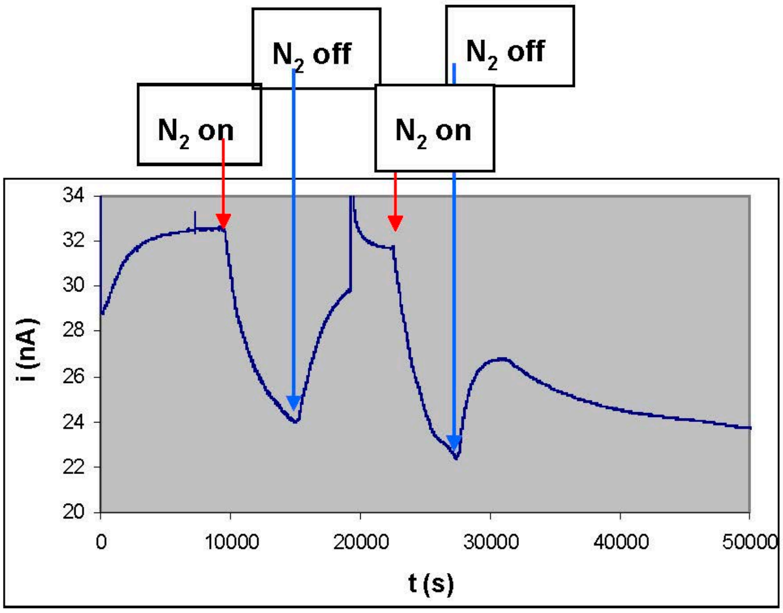

© 2014 by the authors; licensee MDPI, Basel, Switzerland. This article is an open access article distributed under the terms and conditions of the Creative Commons Attribution license (http://creativecommons.org/licenses/by/4.0/).
